# Microplastics increase mercury bioconcentration in gills and bioaccumulation in the liver, and cause oxidative stress and damage in *Dicentrarchus labrax* juveniles

**DOI:** 10.1038/s41598-018-34125-z

**Published:** 2018-10-23

**Authors:** Luís Gabriel Antão Barboza, Luís Russo Vieira, Vasco Branco, Cristina Carvalho, Lúcia Guilhermino

**Affiliations:** 1 ICBAS – Institute of Biomedical Sciences of Abel Salazar, University of Porto, Department of Populations Study, Laboratory of Ecotoxicology & CIIMAR – Interdisciplinary Centre of Marine and Environmental Research, University of Porto, Research Group of Ecotoxicology, Stress Ecology and Environmental Health (ECOTOX), ICBAS - Rua de Jorge Viterbo Ferreira, 228, 4050-313 Porto, Portugal; 2 CAPES Foundation, Ministry of Education of Brazil, 70040-020 Brasília, DF Brazil; 3 Research Institute for Medicines (iMed.ULisboa), Faculty of Pharmacy, Universidade de Lisboa, Av. Prof. Gama Pinto, 1649-003 Lisboa, Portugal

## Abstract

The presence of microplastics and several other pollutants in the marine environment is of growing concern. However, the knowledge on the toxicity of mixtures containing microplastics and other contaminants to marine species is still scarce. The main goals of this study were to investigate the oxidative stress and lipid oxidative damage potentially induced by 96 h of exposure to mercury (0.010 and 0.016 mg/L), microplastics (0.26 and 0.69 mg/L), and mixtures of the two substances (same concentrations, full factorial) in the gills and liver of *D. labrax* juveniles, and the possible influence of microplastics on mercury bioconcentration (gills) and bioaccumulation (liver). The results indicate that the presence of microplastics in the water increased the concentration of mercury in gills and liver of *D. labrax* juveniles. Microplastics and mercury, alone and in mixtures, caused oxidative stress in both organs. Based on the total induction of antioxidant enzymatic activity, the type of toxicological interaction in fish exposed to the mixture containing the lowest concentration of the two substances was addition in gills, and addition or synergism in the liver. These results stress the need to further address the role of microplastics in the bioconcentration, bioaccumulation, and toxicity of other environmental contaminants in different species.

## Introduction

Over the last few years, microplastics have been found in the environment worldwide, including enclosed water bodies and remote areas^1,2^, and are now considered global pollutants of priority study^3–5^. Such particles result either from the fragmentation of larger plastic debris in the environment or from specifically produced micro- or nanosized plastics used for several purposes (e.g. pre-production pellets, cleaning agents, textiles, cosmetics and personal care products)^6^. The levels of microplastics in aquatic environments are diverse, such as 2.46 particles/m^3^ in the Northeast Atlantic Ocean^7^, 0.0032 to 1.18 particles/m^3^ in the Ross Sea (Antarctica)^8^, 0.028 particles/m^3^ in the Tamar Estuary, UK^9^, 300 ng/mL in the North Pacific subtropical gyre^10^, and high abundances and concentrations have been found in polluted areas such as 228 particles m^−2^ in the Coastline of Qatar Gulf^11^, 324 particles/m^3^ or 64,812,600 particles/km^2^ in the Israeli Mediterranean coastal waters^12^, and average concentrations of 1.56 ± 1.64 and 5.51 ± 9.09 mg/L in lakes and wetlands^13^. Data on the microplastics concentration found in the environment are often difficult to compare due to the lack of standardized sampling methodologies, normalization units and expression of data^14^.

Due to their small size, microplastics are in the size range of food particles normally ingested by several aquatic animals^15^. The reasons for the ingestion of these small particles include their accidental consumption by aquatic filter feeders^16^, and active selection (e.g. confusion of microplastics with a prey), since many species are attracted to these microparticles based on their attributes such as shape and color^17,18^ through sensory signals (i.e. visual or olfactory cues)^19^. Microplastics are also ingested indirectly as a result of trophic transfer, when contaminated prey are consumed by their predators^20,21^. After ingestion or after crossing the gills, microplastics absorption and distribution through the circulatory system can occur, and if so the particles may be incorporated into different tissues and cells^22^. This can result in several types of effects, such as: behavior alterations, predatory performance reduction, neurotoxicity, inflammation, hepatic stress, metabolic disorders, decreased growth, among others^23–29^. Moreover, the uptake of microplastics contaminated with other environmental contaminants has been suggested as a possible additional exposure route to several chemicals harmful to aquatic organisms including styrene, metals, phthalates, bisphenol A, polychlorinated biphenyls and polycyclic aromatic hydrocarbons^30,31^. For this reason, the potential for microplastics and associated contaminants to undergo bioaccumulation and trophic transfer is high^15^.

The accumulation of environmental contaminants by microplastics is likely important in ecosystems contaminated with complex mixtures of chemicals such as estuaries impacted by strong industrial, urban and/or agricultural surroundings. This may cause adverse effects on the biota of these systems, including important marine species such as the European seabass *Dicenthrarchus labrax* (Linnaeus, 1758) that spends part of its life cycle within estuaries before reaching maturity^32^. The ingestion of microplastics by *D. labrax* from an estuarine ecosystem was recently reported^33^. In this species, exposure to microplastics can cause several adverse effects, including behavioral changes, intestinal alterations, and neurotoxicity^27–29,34^. Moreover, the exposure of *D. labrax* juveniles to mixtures of microplastics and mercury (another common contaminant of high concern found in different concentrations in the environment such as 0.5 to 200 ng/L in the North Sea^35^, 39 to 430 ng/L in the Wuli Estuary, China^36^, and 990 to 27,060 ng/L in the Mediterranean Sea^37^) was found to reduce the swimming performance, cause neurotoxicity, and induce changes in the activity of energy-related enzymes^27,28^.

To complement these studies, the oxidative stress and lipid oxidative damage potentially induced by 96 h of exposure to mercury (0.010 and 0.016 mg/L), microplastics (0.26 and 0.69 mg/l), and mixtures of the two substances (same concentrations, full factorial) in the gills and liver of *D. labrax* juveniles, and the possible influence of microplastics on mercury bioconcentration (gills) and bioaccumulation (liver) were investigated. In this study, “bioconcentration” was used to refer the direct uptake of microplastics from the water by the gills, whereas “bioaccumulation” was used to indicate the accumulation in the liver after absorption (through all exposure routes), distribution, storage and elimination.

## Results and Discussion

### Mercury concentrations, bioconcentration and bioaccumulation factors, and influence of micro-plastics

The concentrations of mercury (mean ± SD) in gills ranged from 1.519 ± 0.369 μg/g to 4.825 ± 0.881 μg/g, whereas in the liver they ranged from 2.571 ± 0.903 μg/g to 8.169 ± 1.398 μg/g (Table 1). The bioconcentration factors (BCF) in gills ranged from 152 ± 37 to 302 ± 55 and the bioaccumulation factors (BAF) in the liver ranged from 257 ± 86 to 511 ± 80 (Table 1). Thus, fish uptake the metal from the water, bioconcentrate it in gills and accumulate it in the liver. These findings are in good agreement with previous studies reporting accumulation of mercury by *D. labrax*^27,38^.

**Table 1:** Concentrations of mercury (Hg) in *Dicentrarchus labrax* gills and liver (μg/g wet weight), bioconcentration factors (BCF) and bioaccumulation factors (BFA) after 96 hours of exposure.

Treatments	Gills Hg Conc. (µg/g)	Post hoc test	BCF gills	Post hoc test	Liver Hg Conc. (µg/g)	Post hoc test	BAF liver	Post hoc test
Hg low	1.519 (±0.369)	A	152 (±37)	a	3.127 (±0.753)	A	313 (±75)	a
Hg high	2.836 (±0.535)	B	177 (±33)	a,b	5.419 (±1.826)	B	339 (±92)	a
MPs low + Hg low	2.670 (±0.918)	B	267 (±92)	b,c	2.571 (±0.903)	A	257 (±86)	a
MPs low + Hg high	4.310 (±0.965)	C	269 (±60)	b,c	4.370 (±2.296)	A,B	273 (±96)	a
MPs high + Hg low	2.995 (±1.158)	B	300 (±86)	c	5.040 (±1.179)	B	504 (±87)	b
MPs high + Hg high	4.825 (±0.881)	C	302 (±55)	c	8.169 (±1.398)	C	511 (±80)	b

In the columns of concentrations, BCF and BAF, the values are the mean and standard deviation of nine replicates (fish) after discounting the mean of control group. For each data set (i.e. gills or liver mercury concentrations, BCF and BAF) different letters in the post-hoc test columns indicate statistical significant differences (Kruskal-Wallis test + non-parametric multicomparison test, p ≤ 0.05).

Significant differences in the concentrations of mercury among distinct treatments were found for both gills (χ^2^_(5)_ = 36.384, p = 0.000) and liver (χ^2^_(5)_ = 33.084, p = 0.000). Significant differences in gill BCF (χ^2^_(5)_ = 28.066, p = 0.000) and liver BAF (χ^2^_(5)_ = 27.287, p = 0.000) among fish exposed to distinct treatments were also found. In fish exposed to mercury alone, the concentration of metal in both gills and liver was significantly higher in fish exposed to water containing 0.016 mg/L of mercury than in fish exposed to treatments containing 0.010 mg/L of mercury (Table 1). Thus, the accumulation of mercury depends on the water exposure concentration. The comparison of the BCF and BAF factors obtained in the present study in fish exposed to mercury alone (Table 1) with those determined previously in brain (BAF = 5 and 7) and muscle (BAF = 28 and 40) tissues^27^ indicates the following decreasing order of mercury accumulation or bioconcentration in tissues of *D. labrax* juveniles: liver > gills > muscle > brain.

**Figure 1:**
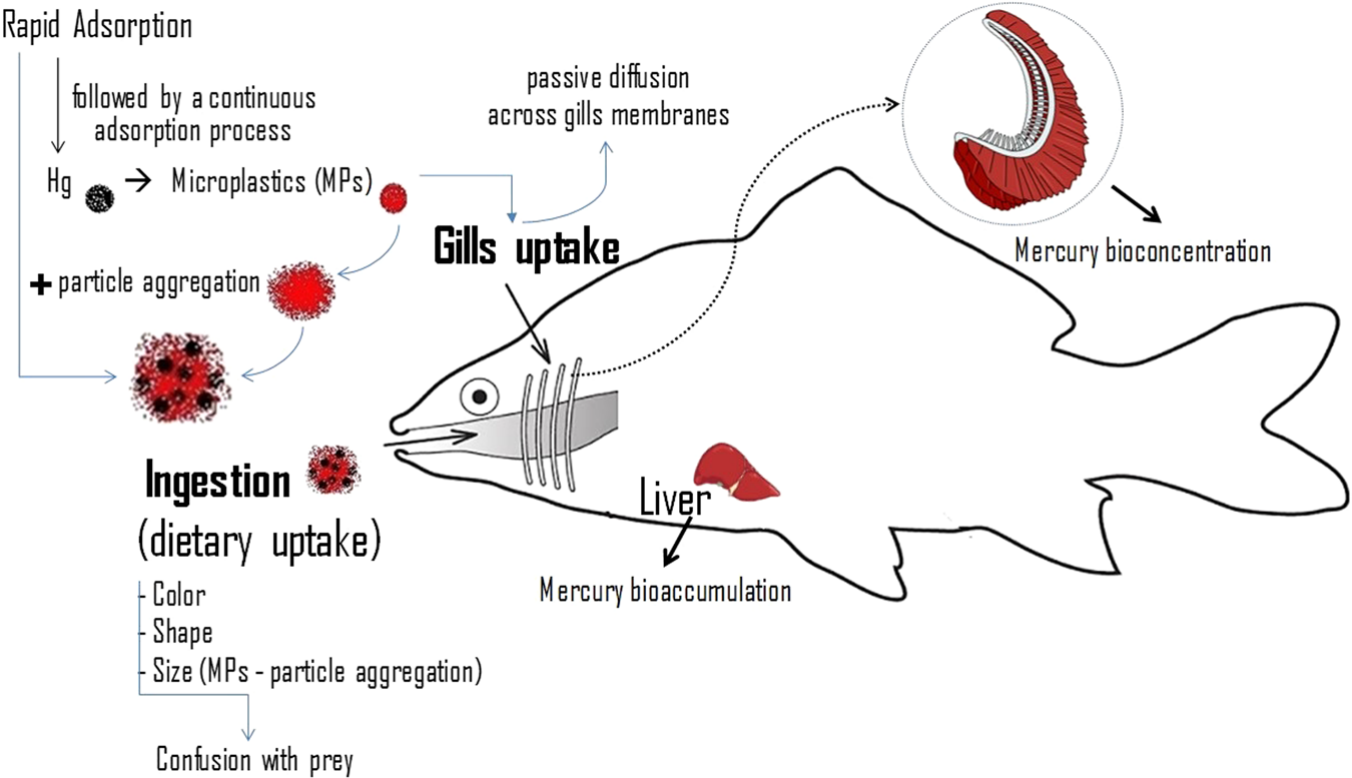
Potential influence of microplastics on mercury bioconcentration and bioaccumulation by fish.

Fish exposed to the metal alone had significantly lower mercury concentrations in gills than those exposed to the same concentration of mercury in combination with microplastics (Table 1). In the liver, a comparable situation occurred, but only in relation to the highest concentration of mercury tested (Table 1). Thus, the presence of microplastics had influence on the mercury concentrations in gills and liver. Such influence of microplastics may have been due to several processes. For example (Fig. 1), microplastics may absorb mercury from the water and act as an additional exposure route to the metal. Because microplastics are frequently stocked in gills of aquatic animals^5,39^, if the microplastics uptaken by fish though the gills had mercury adsorbed this could have result in increased concentrations of the metal in the gills exposed to the mixtures. Moreover, in the gills, release of the metal from the particles and absorption of at least part of it may have occurred leading to increased accumulation of mercury also in other organs such as the liver. A comparable process may have occurred in the digestive system (Fig. 1) also contributing to increase the mercury concentrations in the liver. Previous studies indicating that mercury absorbs to microplastic virgin pellets provide support to this hypothesis^40^. In addition to the processes discussed above, the presence of microplastics in the gills may have interfered with the mechanisms regulating the uptake and elimination of the metal locally. Additionally, the presence of the particles in the gills may have decreased the oxygen uptake leading to hypoxia, subsequent reduction of the aerobic cellular energy production, as hypothesized for *Daphnia magna* exposed to the same type of microplastics^41^. If so, the elimination of mercury may have been reduced in fish exposed to mixtures due to shortage of energy available.

### Oxidative stress and damage induced by microplastics, mercury and their mixtures

**Figure 2:**
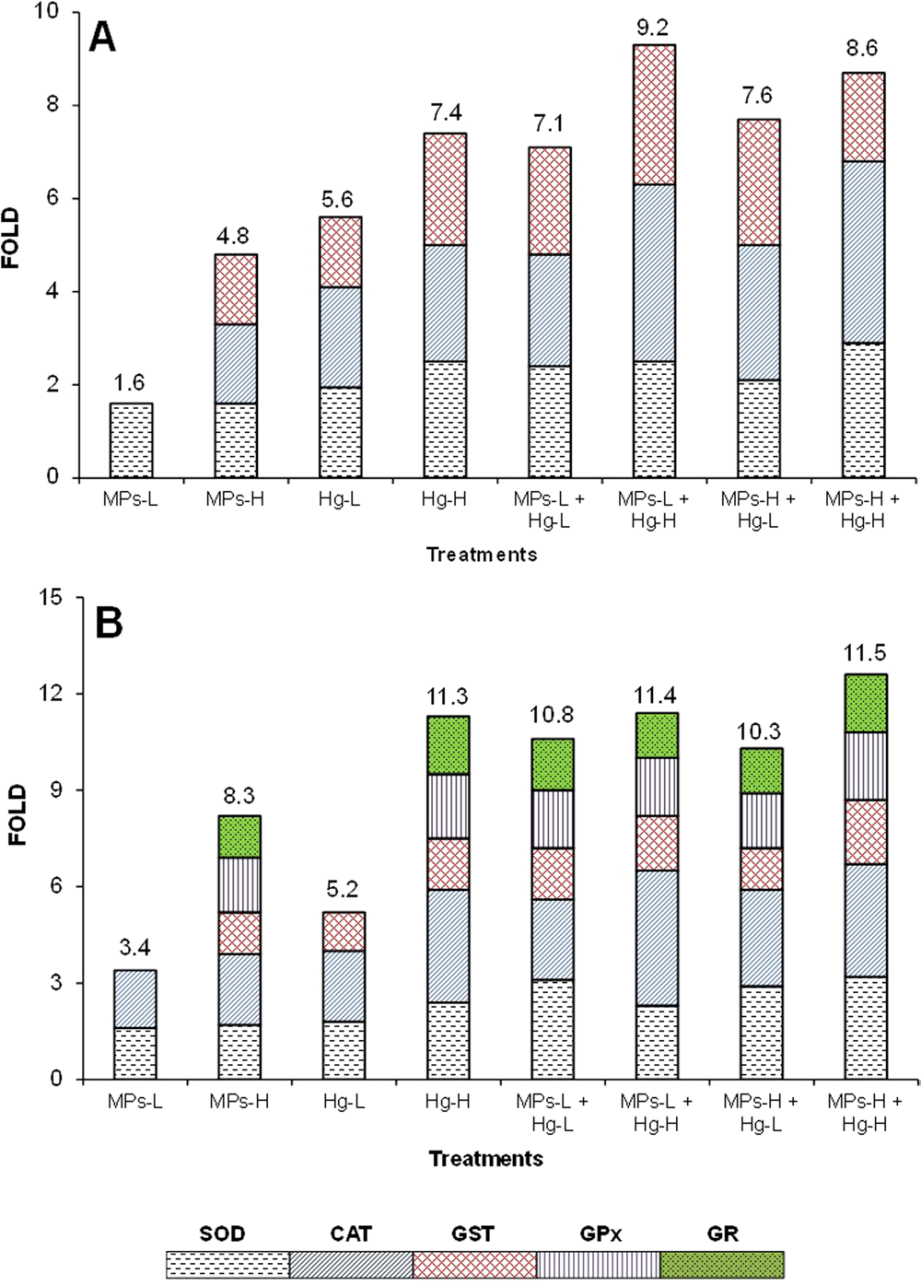
Contribution of enzymes superoxide dismutase (SOD), catalase (CAT), glutathione S-transferase (GST), glutathione peroxidase (GPx) and glutathione reductase (GR) in the antioxidant defense system of *Dicentrarchus labrax (***A** – gills; **B** – liver). Numbers above the columns indicate the total induction (fold).

Significant differences (p ≤ 0.05) in all the oxidative stress and damage biomarkers among treatments were found in both gills and liver (complete results in Table S-1, supplementary information). The anti-oxidant enzymes with significantly increased activity are shown in Fig. 2.

**Figure 3:**
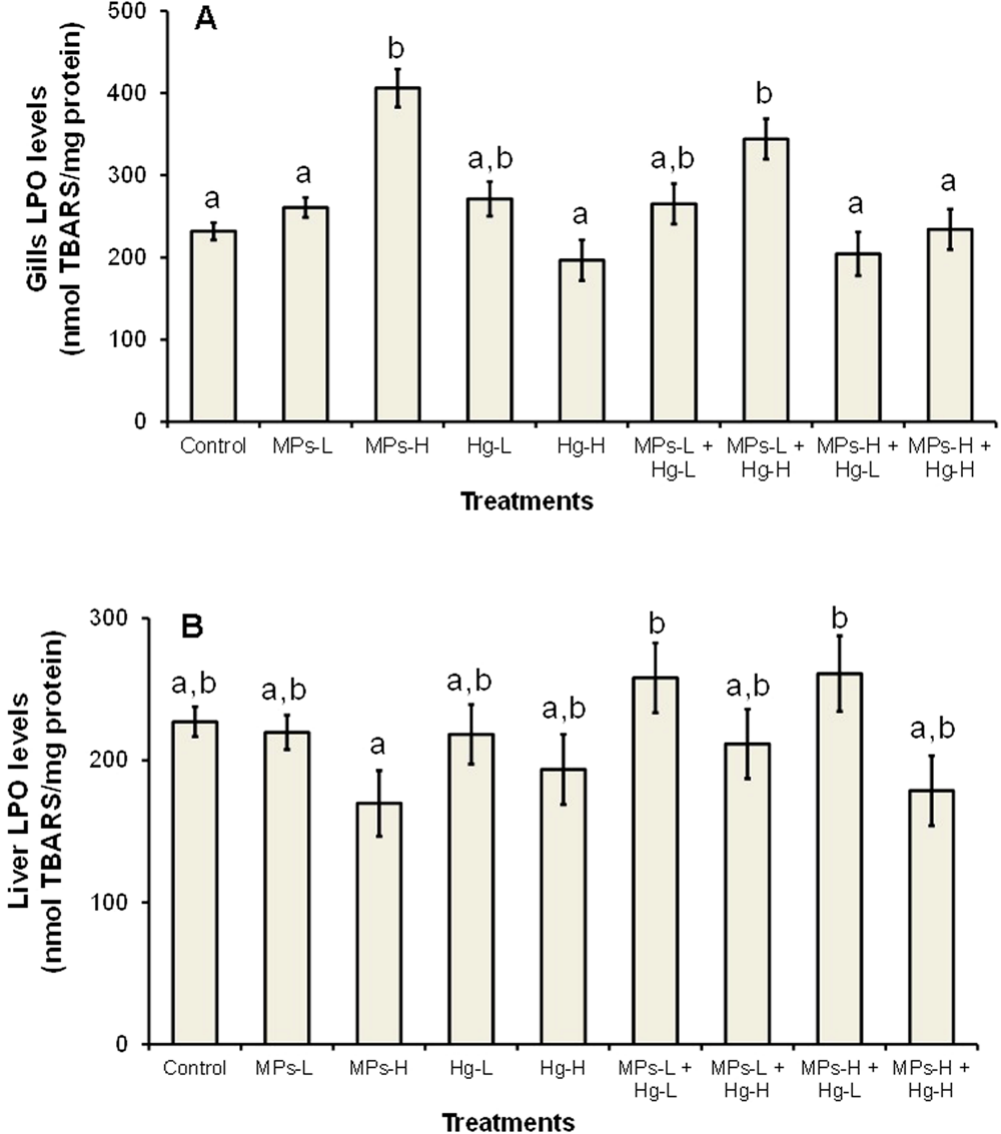
Gills (**A**) and liver (**B**) lipid peroxidation (LPO) in *Dicentrarchus labrax* exposed for 96 h to microplastics (MPs), mercury (Hg) or mixtures of the two substances. The values are the mean per treatment (9 animals) with corresponding standard error bars (SEM). Different letters indicate statistically significant differences between treatments (p < 0.05, Tukey test).

In relation to the control group, fish exposed to 0.26 mg/L of microplastics alone had significantly increased superoxide dismutase (SOD) activity (1.6-fold) in gills (Fig. 2A), and significantly increased SOD and catalase (CAT) activities (3.4-fold of total anti-oxidant enzymatic induction, hereafter indicated as total induction) in the liver (Fig. 2B). The induction of these anti-oxidant enzymes was probably enough to cope with the oxidative stress induced by the lowest concentration of microplastics tested because no significant increase of lipid peroxidation (LPO) levels was observed (Fig. 3). Fish exposed to the highest concentration of microplastics alone (0.69 mg/L), had significant induction of CAT, glutathione-S-transferase (GST) and SOD, resulting in a total induction of 4.8-fold. Despite the induction of two additional enzymes, the LPO levels were significantly increased (Fig. 3A) indicating that lipid oxidative damage in gills occurred. In the liver, fish exposed to 0.69 mg/L of microplastics alone, had significantly induced activities of SOD, CAT, GST, glutathione peroxidase (GPx) and glutathione reductase (GR), resulting in a total induction of 8.3-fold which was enough to avoid lipid oxidative damage in this organ (Fig. 3B). Overall, these results indicate that microplastics induced oxidative stress in both gills and liver at concentrations ≥0.26 mg/L and lipid oxidative damage in gills at 0.69 mg/L. This may have been caused by indirect effects resulting from physical damage caused by the particles themselves and/or by additives that the microplastics likely contain. The microplastics-induced oxidative stress and damage found here are in agreement with the microplastic-induced oxidative stress and damage in brain and muscle of *D. labrax* juveniles previously described^27^. Oxidative stress induced by different types of microplastics was also reported in other species, such as the fish *Danio rerio*^42^, the bivalves *Scrobicularia plana*^43^ and *Corbicula fluminea*^5^, and the rotifer *Brachionus koreanus*^44^.

In relation to the control group, fish exposed to the lowest concentration of mercury alone (0.010 mg/L) showed significant induction of SOD, CAT and GST activities in both gills and liver, in a total induction of 5.6 and 5.2-fold, respectively (Fig. 2A,B), and no significant changes in LPO levels (Fig. 3A,B). Exposure to 0.016 mg/L of mercury alone resulted in a higher induction of SOD, CAT and GST activities in gills (total induction of 7.4-fold). In the liver, mercury exposure caused the additional induction of GPx and GR activities, with a total induction of 11.3-fold (Fig. 2B). In both organs, no significant increase of LPO levels occurred (Fig. 3). Therefore, exposure to mercury (0.010 mg/L and 0.016 mg/L) caused oxidative stress in *D. labrax* juveniles but did not result in lipid oxidative damage. Oxidative stress is a well-known effect of mercury previously reported in *D. labrax*^27,38^ and other fish species^45–47^.

All the mixtures tested induced the activity of three anti-oxidant enzymes in gills (SOD, CAT and GST) and five in the liver (SOD, CAT, GPx, GR and GST) (Fig. 2). The mixture containing the lowest concentration of microplastics and the highest concentration of mercury also caused a significant increase of LPO levels in gills (Fig. 3A), suggesting toxicological interactions between the two substances in *D. labrax* juveniles. Thus, with the exception of this mixture, the induction of anti-oxidant enzymes was likely enough to prevent the occurrence of lipid oxidative damage. The results of 2-ANOVA (complete results in Table S-2, supplementary information) carried out with some gills (CAT, GPx, GST and LPO) and liver (SOD, CAT, GST and LPO) biomarkers, also indicated significant interaction (p ≤ 0.05) between microplastics and mercury suggesting toxicological interactions between microplastics and mercury in *D. labrax* juveniles. Moreover, in gills, the total induction of anti-oxidant enzymatic activity caused by the mixture containing the lowest concentrations of microplastics and mercury tested (7.1-fold) was comparable to the sum of the total induction caused by the same concentrations of the substances individually (1.6 + 5.6 = 7.2-fold). In the liver, the same mixture induced a higher total induction (10.8-fold) than the sum of the total induction caused by microplastics and mercury individually (3.4 + 5.2 = 8.6-fold). These results suggest that the type of toxicological interaction may be addition in gills, and addition or synergism in the liver. At higher concentrations of one or both mixture components it was not possible to draw conclusions about the type of interaction because, after a certain level, the induction of anti-oxidant enzymes does not necessary increase with the increase of the exposure concentrations. This is a well-known behaviour of anti-oxidant enzymes towards a high number of environmental contaminants that is often indicated as “bell-shape behaviour”^45,48^.

## Conclusions

The concentrations of mercury in both gills and liver of *D. labrax* juveniles were significantly higher in the presence of microplastics than in their absence, indicating that microplastics influence the bioconcentration of the metal in gills and its bioaccumulation in the liver. The concentrations of microplastics and mercury tested, alone and in mixture, caused oxidative stress in gills and liver of *D. labrax* juveniles. Additionally, the highest concentration of microplastics caused lipid oxidative damage in gills. In fish exposed to mixtures, evidence of toxicological interactions between microplastics and mercury were found. At low concentrations of both mixture components and based on the total induction of anti-oxidant enzymes activity, the type of toxicological interaction likely is addition in gills, and addition or synergism in the liver. These findings stress the need of further investigating the influence of microplastics in the bioconcentration, bioaccumulation, absorption, elimination and toxicity of other environmental contaminants in different species.

## Material and Methods

### Chemicals

Fluorescent red polymer microspheres (1–5 μm diameter) were used as microplastics particles and were purchased from Cospheric – Innovations in Microtechnology (USA). According to manufacturer indications, 1 mg of the product contains about 1.836E + 8 spheres (estimate made for an average of 2 μm diameter). Mercury chloride (≥99.5% pure) was purchased from Sigma-Aldrich (USA). The Bradford reagent used for protein determinations was from BIORAD (Germany). All the other chemicals for biomarkers determinations were of the highest purity available and purchased from Sigma-Aldrich (USA) or Merck (Germany).

### Ethical issues

Experiments were authorized by the Portuguese National Authority for Animal Health (“Direção Geral de Agricultura e Veterinária” - DGAV) and conducted according to the ethical principles and other requirements of Portuguese and EU regulations for the protection of animals used for scientific purposes. L. Guilhermino and L. R. Vieira are accredited by the DGAV as investigator/coordinator (equivalent to FELASA category C) to carry animal experimentation. The experiments were carried out in the CIIMAR bioterium, which is accredited by DGAV for studies with aquatic animals.

### Bioassay

**Figure 4:**
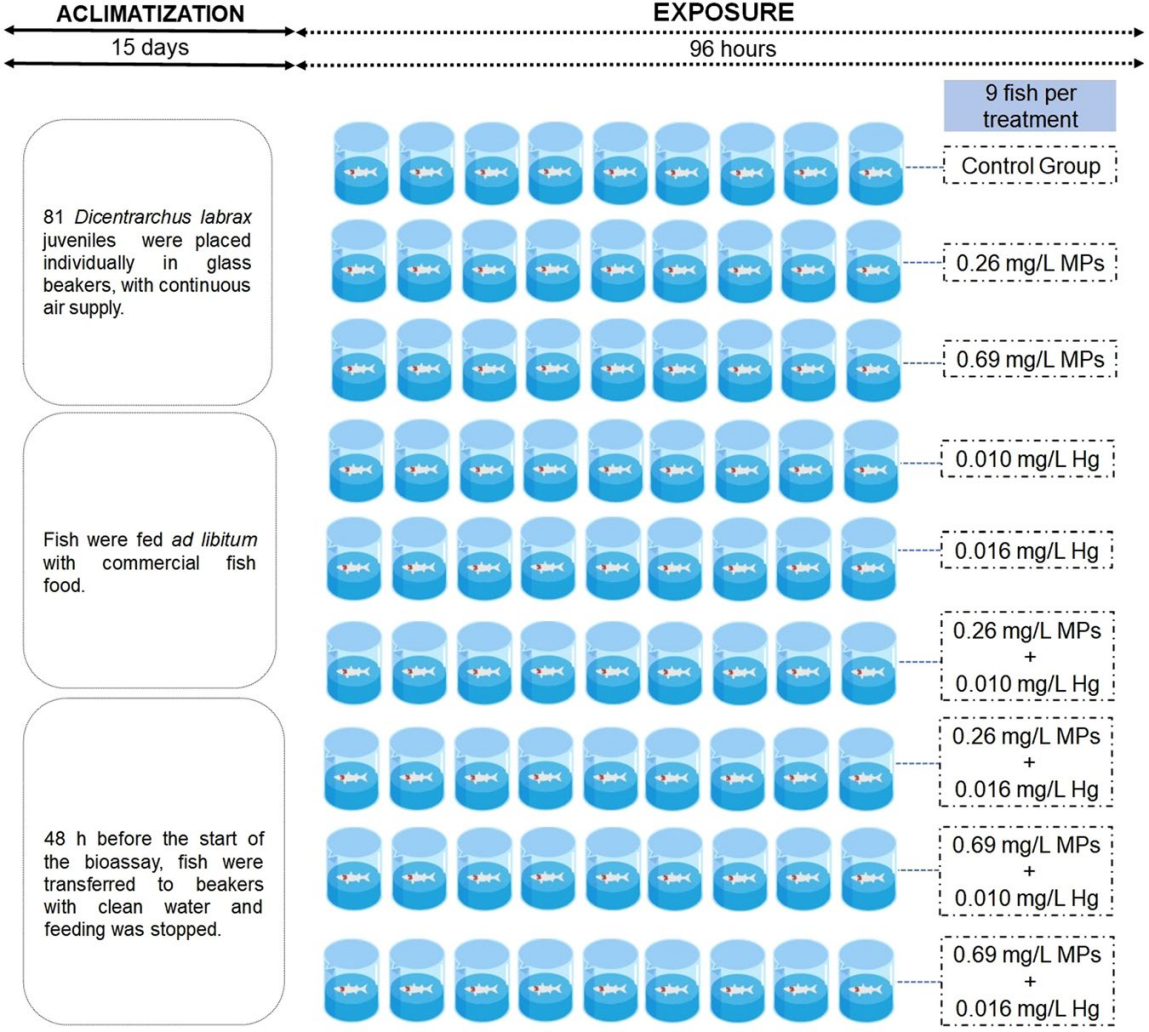
Experimental design scheme.

The test species, *Dicentrarchus labrax*, was selected for this study because of its wide use for human consumption, high commercial value, important ecological functions, and wide use in ecotoxicological studies^49,50^. The juveniles used were measured at (mean ± standard deviation) 7.75 ± 0.293 cm (total length) and 8.82 ± 0.295 g (body wet weight – w.w.). The experimental design, fish exposure and tissue isolation are described in detail in Barboza *et al*.^27^. Briefly, fish purchased from an aquaculture were acclimatized to laboratory conditions in a room with controlled temperature and photoperiod (19 ± 1 °C, photoperiod: 14 h light: 10 h dark), in UV-filtered seawater (salinity: 34 ± 1 gL^−1^). After this period, 81 *D. labrax* juveniles were randomly distributed per 9 treatments (9 fish per treatment). Our schematic procedure of experiment is shown in Fig. 4. The exposure period was 96 h and no food was provided to fish during the experiment. Test beakers were glass, filled with 4 L of filtered water and continuous additional air supply. Water was renewed (*i.e*. completely replaced) every 24 h. Water samples for determination of mercury and microplastics concentrations were collected at the beginning and the end of the bioassay and at each water renewal, including the collection of both clean and old water. Water samples were stored at −20 °C until further analyses. After 96 h of exposure, samples of gills and liver were collected from each fish as indicated in Barboza *et al*.^20^ and stored at −80 °C. Both concentration of microplastics and both concentrations of mercury tested are ecologically relevant^10,13,37^. The higher concentration of microplastics tested (0.69 mg/L) is lower than those reported for some polluted waters^13^.

### Biomarkers determination

Several biomarkers involved in important physiological functions related to fish health status maintenance were measured, namely gill and liver superoxide dismutase (SOD) activity, gill and liver catalase (CAT) activity, gill and liver glutathione peroxidase (GPx) activity, gill and liver glutathione reductase (GR) activity, gill and liver glutathione-S-transferase (GST) activity and gill and liver lipid peroxidation (LPO) levels. Antioxidant enzymes including SOD, CAT, GPx, GR and GST were selected because they usually act in a coordinated manner in order to ensure the optimal protection against oxidative stress. LPO levels were selected as marker of oxidative damage to lipids. On the day of the analyses, liver and gill samples (1:10 g wt v^−1^) were homogenized in phosphate buffer (pH 7.4, 0.1 M). Homogenates were divided into aliquots to analyse LPO and total mercury concentration. One aliquot was used for enzymatic activity assays following post-mitochondrial fraction isolation (centrifugation for 20 min at 10,000 g at 4 °C). All biomarkers and protein determinations were made at 25 °C. The protein content of the samples was determined by the Bradford method^51^ adapted to microplate^52^. Then, it was standardized to 0.3 mg mL^−1^ (GST samples) or to 1 mg mL^−1^ (LPO, SOD, CAT, GPx and GR samples). LPO levels were determined by quantification of thiobarbituric acid reactive substances (TBARS) at 535 nm^53^. GST activity was determined at 340 nm^54^ adapted to microplate^55^. SOD, GPx, GR activities were determined by the techniques of Flohé and Ötting^56^, Flohé and Gunzler^57^ and Carlberg and Mannervik^58^, respectively, with adaptations^59^. CAT activity was determined according to Clairborne^60^ at 240 nm. All analyses were performed in a Spectramax® spectrophotometer (Molecular Devices, USA). LPO levels were expressed in nanomoles of TBARS per mg of protein (nmol TBARS/mg protein). SOD activity was expressed in one unit per mg of protein (U/mg protein). CAT activity was expressed in micromoles per mg of protein (µmol/min/mg protein). GPx, GR and GST activities were expressed in nanomoles per mg of protein (nmol/min/mg protein).

### Mercury concentrations and bioaccumulation factors

The preparation of water and tissue samples for mercury analyses is described in detail in Barboza *et al*.^27^. Briefly, water samples containing microplastics were filtered with a nylon membrane syringe filter with a pore size of 0.2 μm (Acrodisc®) and stored in Teflon tubes for further analysis. Liver and gills samples were thawed individually, agitated for 1 min in a vortex mixer, after which 0.100 mL were collected for analysis. Mercury concentrations in water and tissues samples were determined by atomic absorption spectrometry (AAS) using a silicon UV diode detector (AMA-254, LECO, Czech Republic) as described in detail in Barboza *et al*.^27^. The accuracy of the analytical procedure was verified through the analysis of a certified reference material (CRM), BCR 463 (mercury and methyl-mercury in tuna fish). The mercury bioconcentration factors (BCF) and mercury bioaccumulation factors (BAF) were determined according to Beldowska and Falkowska^61^ as: BCF = mercury concentration in the gills (ppm)/mercury concentration in the water (ppm); BAF = mercury concentration in the liver (ppm)/mercury concentration in the water (ppm). The mercury concentrations in the water are given in detail in Barboza *et al*.^27^ and according to these results the mean water ± SD exposure concentrations during the interval of water renewal were 0.010 ± 0.0008 mg/L and 0.016 ± 0.0009 mg/L in treatments with the lowest and the highest mercury concentrations, respectively. Mean values were used to calculate the BCF and BAF factors in fish exposed to treatments containing the lowest or the highest mercury concentrations, respectively.

Water microplastics concentrations were determined in clean and old water by spectrofluorimetry following Luís *et al*.^62^, with adaptations to the type of water and microplastics used^17^. Between water renewals (every 24 h), the mean (±SD) microplastic exposure concentration was 0.26 ± 0.028 mg/L and 0.69 ± 0.036 mg/L in treatments containing the lowest and the highest concentrations of the particles, respectively^27^.

### Statistical analyses of data

Statistical analyses were performed using the SPSS statistical analysis package (version 24.0). For each data set, normality of distribution and equality of variance were checked by Shapiro-Wilk test and Levene’s test, respectively. When these assumptions were not fulfilled, Analysis of Variance (ANOVA) was preceded by data transformation^63^. Each data set was analysed through one-way ANOVA (1-ANOVA) or two-way ANOVA with interaction (2-ANOVA) followed by the Tukey’s multiple comparisons test when statistical significant differences were found. When ANOVA assumptions could not be achieved even after data transformation, the non-parametric Kruskal-Wallis test was used, followed by a nonparametric multiple comparisons test (using Dunn’s procedure with a Bonferroni adjustment when significant differences were found). Differences between treatments were considered significant a p-level < 0.05.

## Electronic supplementary material

Supplementary Information
