# Supplementary material for: Microplastics increase mercury bioconcentration in gills and bioaccumulation in the liver, and cause oxidative stress and damage in Dicentrarchus labrax juveniles
Source: Sci Rep. 2018 Oct 23;8:15655. doi: 10.1038/s41598-018-34125-z (PMC6199270; doi:10.1038/s41598-018-34125-z)
Supplement: Supplementary file 1 — Supplementary Information [file 41598_2018_34125_MOESM1_ESM.doc]

|  |  |
| --- | --- |

**Supplementary Information**

**Microplastics increase mercury bioconcentration in gills and bioaccumulation in the liver, and cause oxidative stress and damage in *Dicentrarchus labrax* juveniles**

Luís Gabriel Antão Barboza*^1,2^, Luís Russo Vieira^1^, Vasco Branco^3^, Cristina Carvalho^3^, Lúcia Guilhermino^1^

1. ICBAS – Institute of Biomedical Sciences of Abel Salazar, University of Porto, Department of Populations Study, Laboratory of Ecotoxicology & CIIMAR – Interdisciplinary Centre of Marine and Environmental Research, University of Porto, Research Group of Ecotoxicology, Stress Ecology and Environmental Health (ECOTOX). ICBAS - Rua de Jorge Viterbo Ferreira, 228, 4050-313 Porto, Portugal.
2. CAPES Foundation, Ministry of Education of Brazil, 70040-020, Brasília – DF, Brazil.
3. Research Institute for Medicines (iMed.ULisboa), Faculty of Pharmacy, Universidade de Lisboa, Av. Prof. Gama Pinto, 1649-003 Lisboa, Portugal.

Correspondence and requests for materials should be addressed to L.G.A.B (email: lbarboza@ciimar.up.pt).

This Supplementary Information contains:

| **Page S2:** | Results of the one-way ANOVA or Kruskal-Wallis indicating significant differences among treatments for all biomarkers (p ≤ 0.05). **Table S-1.** |
| --- | --- |
| **Page S3:** | Results of the two-way Analysis of Variance with interaction investigating the effects of microplastics and mercury on several biological parameters of *Dicentrarchus labrax* comparing the effects of microplastics concentration and mercury concentration on the effects criteria at the end of the exposure period (96 hours). Fixed factors: microplastics concentrations (0.26 mg/L and 0.69 mg/L) and mercury concentrations (0.010 mg/L and 0.016 mg/L). SD – standard deviation, **Table S-2** |

|  |  |
| --- | --- |
|  |  |

|  | **Table S-1.** Results of the one-way ANOVA or Kruskal-Wallis indicating significant differences among treatments for all biomarkers (p ≤ 0.05).   | **Biomarker** | **Mean ± SD** | **Statistical analyses** | | --- | --- | --- | | **SOD (gills)** | 28.039 ± 9.053 | χ^2^_(8)_ = 56.805; p = 0.000 | | **CAT (gills)** | 211.912 ± 23.913 | F_(8,72)_ =108.339; p = 0.000 | | **GPx (gills)** | 5.383 ± 2.082 | F_(8,72)_ = 2.846; p = 0.008 | | **GR (gills)** | 10.443 ± 2.769 | χ^2^_(8)_ = 23.368; p = 0.000 | | **GST (gills)** | 101.508 ± 39.485 | F_(8,72)_ = 52.798; p = 0.000 | | **LPO (gills)** | 267.212 ± 142.743 | F_(8,72)_ = 2.703; p = 0.012 | | **SOD (liver)** | 31.491 ± 11.944 | F_(8,72)_ = 22.909; p = 0.000 | | **CAT (liver)** | 42.384 ± 17.676 | F_(8,72)_ = 23.275; p = 0.000 | | **GPx (liver)** | 24.226 ± 7.534 | χ^2^_(8)_ = 48.558; p = 0.000 | | **GR (liver)** | 19.919 ± 5.227 | χ^2^_(8)_ = 58.735; p = 0.000 | | **GST (liver)** | 163.908 ± 39.480 | F_(8,72)_ = 39.613; p = 0.000 | | **LPO (liver)** | 215.289 ± 61.875 | F_(8,72)_ = 2.767; p = 0.010 |   **Table S-2.** Results of the two-way Analysis of Variance with interaction investigating the effects of microplastics and mercury on several biological parameters of *Dicentrarchus labrax* comparing the effects of microplastics concentration and mercury concentration on the effects criteria at the end of the exposure period (96 hours). (SOD and GR in the gills and GPx and GR in the liver could not be analysed because the ANOVA assumptions were not fulfilled). Fixed factors: microplastics concentrations (0.26 mg/L and 0.69 mg/L) and mercury concentrations (0.010 mg/L and 0.016 mg/L). SD – standard deviation.   | **Parameter** | **Factor** | **Level**  **(mg/L)** | **Mean ± SD** | **Tukey test** | **F** | **p** |  | | | --- | --- | --- | --- | --- | --- | --- | --- | --- | | **Gills CAT** | MPs | 0 | 103.254 ± 37.679 | a | F_(2.72)_ =5.445 | 0.000 |  | | | 0.26 | 128.560 ± 67.097 | b |  | | | 0.69 | 153. 370 ± 53.731 | c |  | | | Hg | 0 | 65.127 ± 19.69 | A | F_(2.72)_ = 283.558 | 0.000 |  | | | 0.010 | 135. 295 ± 25.180 | B |  | | | 0.016 | 184. 761 ± 39.820 | C |  | | | MPs x Hg |  | |  | F_(4.72)_ = 7.562 | 0.000 |  | | | **Gills GPx** | MPs | 0 | 5.111 ± 2.100 | a,b | F_(2.72)_ = 4.689 | 0.012 |  | | | 0.26 | 6.281 ± 1.866 | b |  | | | 0.69 | 4.757 ± 2.030 | a |  | | |  |  |  |  |  |  |  | | | Hg | 0 | 5.894 ± 1.858 | A | F_(2.72)_ = 4.999 | 0.009 |  | | | 0.010 | 4.433 ± 2.074 | B |  | | | 0.016 | 5.821 ± 2.043 | A |  | | | MPs x Hg |  |  |  | F_(4.72)_ = 0.847 | 0.500 |  | | | **Gills GST** | MPs | 0 | 86. 640 ± 33.019 | a | F_(2.72)_ = 18.088 | 0.000 |  | | | 0.26 | 110.739 ± 49.172 | b |  | | | 0.69 | 107.143 ± 30.624 | b |  | | | Hg | 0 | 61.541 ± 15.074 | A | F_(2.72)_ = 132.543 | 0.000 |  | | | 0.010 | 115.130 ± 32.150 | B |  | | | 0.016 | 127.851 ± 31.049 | C |  | | | MPs x Hg |  |  |  | F_(4.72)_ =30.282 | 0.000 |  | | | **Gills LPO** | MPs | 0 | 233.47 ± 109.731 | a | F_(2.72)_ = 1.663 | 0.197 |  | | | 0.26 | 290.170 ± 148.530 | a |  | | | 0.69 | 281.695 ± 136.53 | a |  | | | Hg | 0 | 299.786 ± 157.793 | A | F_(2.72)_ = 1.367 | 0.261 |  | | | 0.010 | 247.079 ± 106.125 | A |  | | | 0.016 | 258.475 ± 133.667 | A |  | | | MPs x Hg |  | |  | F_(4.72)_ = 3.890 | 0.006 |  | | | **Liver SOD** | MPs | 0 | 24.572 ± 9.106 | a | F_(2.72)_ = 23.891 | 0.000 |  | | | 0.26 | 33.039 ± 11.769 | b |  | | | 0.69 | 36.863 ± 11.633 | b |  | | | Hg | 0 | 20.355 ± 5.713 | A | F_(2.72)_ = 56.178 | 0.000 |  | | | 0.010 | 37.152 ± 10.476 | B |  | | | 0.016 | 36.967 ± 10.166 | B |  | | | MPs x Hg |  | |  | F_(4.72)_ = 5.783 | 0.000 |  |  | | **Liver CAT** | MPs | 0 | 35.197 ± 19.747 | a | F_(2.72)_ = 10.853 | 0.000 |  | | | 0.26 | 45.558 ± 18.423 | b |  | | | 0.69 | 46.397 ± 12.298 | b |  | | | Hg | 0 | 26.760 ± 10.514 | A | F_(2.72)_ = 74.566 | 0.000 |  | | | 0.010 | 41.015 ± 11.630 | B |  | | | 0.016 | 59.377 ± 12.661 | C |  | | | MPs x Hg |  | |  | F_(4.72)_ = 3.840 | 0.007 |  | | | **Liver GST** | MPs | 0 | 145.609 ± 31.986 | a | F_(2.72)_ = 21.531 | 0.000 |  | | | 0.26 | 170.920 ± 37.142 | b |  | | | 0.69 | 175.193 ± 43.154 | b |  | | | Hg | 0 | 130.081 ± 21.150 | A | F_(2.72)_ = 116.184 | 0.000 |  | | | 0.010 | 157.984 ± 29.879 | B |  | | | 0.016 | 203.658 ± 24.296 | C |  | | | MPs x Hg |  | |  | F_(4.72)_ =10.369 | 0.000 |  | | | **Liver LPO** | MPs | 0 | 212.977 ± 64.797 | a | F_(2.72)_ = 1.507 | 0.228 |  | | | 0.26 | 229.772 ± 59.533 | a |  | | | 0.69 | 203.119 ± 60.499 | a |  | | | Hg | 0 | 205.484 ± 66.116 | A | F_(2.72)_ = 6.045 | 0.004 |  | | | 0.010 | 245.811 ± 54.539 | B |  | | | 0.016 | 194.572 ± 54.050 | A |  | | | MPs x Hg |  | |  | F_(4.72)_ = 1.758 | 0.147 |  | | |
| --- | --- | --- | --- | --- | --- | --- | --- | --- | --- | --- | --- | --- | --- | --- | --- | --- | --- | --- | --- | --- | --- | --- | --- | --- | --- | --- | --- | --- | --- | --- | --- | --- | --- | --- | --- | --- | --- | --- | --- | --- | --- | --- | --- | --- | --- | --- | --- | --- | --- | --- | --- | --- | --- | --- | --- | --- | --- | --- | --- | --- | --- | --- | --- | --- | --- | --- | --- | --- | --- | --- | --- | --- | --- | --- | --- | --- | --- | --- | --- | --- | --- | --- | --- | --- | --- | --- | --- | --- | --- | --- | --- | --- | --- | --- | --- | --- | --- | --- | --- | --- | --- | --- | --- | --- | --- | --- | --- | --- | --- | --- | --- | --- | --- | --- | --- | --- | --- | --- | --- | --- | --- | --- | --- | --- | --- | --- | --- | --- | --- | --- | --- | --- | --- | --- | --- | --- | --- | --- | --- | --- | --- | --- | --- | --- | --- | --- | --- | --- | --- | --- | --- | --- | --- | --- | --- | --- | --- | --- | --- | --- | --- | --- | --- | --- | --- | --- | --- | --- | --- | --- | --- | --- | --- | --- | --- | --- | --- | --- | --- | --- | --- | --- | --- | --- | --- | --- | --- | --- | --- | --- | --- | --- | --- | --- | --- | --- | --- | --- | --- | --- | --- | --- | --- | --- | --- | --- | --- | --- | --- | --- | --- | --- | --- | --- | --- | --- | --- | --- | --- | --- | --- | --- | --- | --- | --- | --- | --- | --- | --- | --- | --- | --- | --- | --- | --- | --- | --- | --- | --- | --- | --- | --- | --- | --- | --- | --- | --- | --- | --- | --- | --- | --- | --- | --- | --- | --- | --- | --- | --- | --- | --- | --- | --- | --- | --- | --- | --- | --- | --- | --- | --- | --- | --- | --- | --- | --- | --- | --- | --- | --- | --- | --- | --- | --- | --- | --- | --- | --- | --- | --- | --- | --- | --- | --- | --- | --- | --- | --- | --- | --- | --- | --- | --- | --- | --- | --- | --- | --- | --- | --- | --- | --- | --- | --- | --- | --- | --- | --- | --- | --- | --- | --- | --- | --- | --- | --- | --- | --- | --- | --- | --- | --- | --- | --- | --- | --- | --- | --- | --- | --- | --- | --- | --- | --- | --- | --- | --- | --- | --- | --- | --- | --- | --- | --- | --- | --- | --- | --- | --- | --- | --- | --- | --- | --- | --- | --- | --- | --- | --- | --- | --- | --- | --- | --- | --- | --- | --- | --- | --- | --- | --- | --- | --- | --- | --- | --- | --- | --- | --- | --- | --- | --- | --- | --- | --- | --- | --- | --- | --- | --- | --- | --- | --- | --- | --- | --- | --- | --- | --- | --- | --- | --- | --- | --- | --- | --- | --- |
